# Supplementary figures and images for: Novel and effective plasmid transfection protocols for functional analysis of genetic elements in human cardiac fibroblasts
Source: PLoS One. 2024 Nov 26;19(11):e0309566. doi: 10.1371/journal.pone.0309566 (PMC11594401; doi:10.1371/journal.pone.0309566)

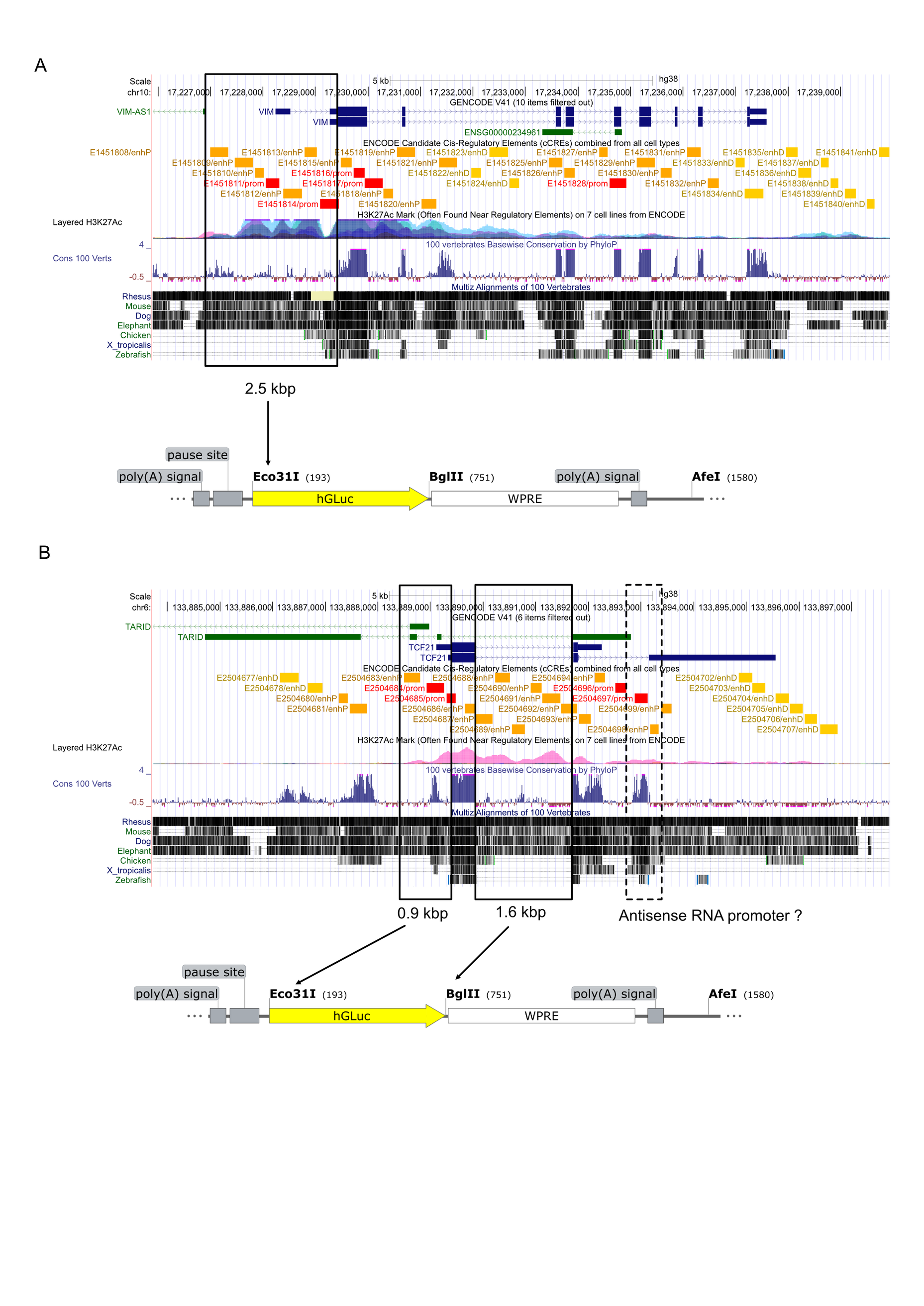

Supplement: S1 Fig — The human Vim (A) and Tcf21 (B) genomic locus with H3K27Ac Mark on seven cell lines from ENCODE, alignments of 100 vertebrates, and ENCODE Candidate Cis-Regulatory Elements (cCREs) combined from all cell types. cCREs are labeled according to the regulatory signatures: Red box, promoter-like signature; orange box, proximal enhancer-like signature; yellow box, distal enhancer-like signature. All data was extracted from USCS Genome Browser. (A) Construction strategy of pGL4.14(Vim, GLuc-WPRE-SpA). Two Vim mRNA sequenes (ENST00000544301.7 and ENST00000224237.9) are registered and there are two promoter-like signatures (red box) in cCREs. PCR 2.5 kbp including both promoter-like signatures, first exon and intron of ENST00000544301.7, second exon of ENST00000544301.7 (this is a first exon of ENST00000224237.9), and the annotated translation start site (TISS), then insert into Eco31I site of a pGL4.14 reporter vector. The secreted luciferase GLuc is expressed downstream of the inserted enhancer/promoter candidates. In addition, synthetic polyadenylation signal and RNA polymerase II transcriptional pause signal from the human α2 globin gene are upstream of the candidate sequences to suppress transcriptions from the vector backbone Amp and ori. (B) Construction strategy of pGL4.14(Tcf21, GLuc-WPRE-SpA). Two Tcf21 mRNAs (ENST00000367882.5 and ENST00000237316.3) are registered and two antisense RNA (TARID, ENST00000607033.5 and ENST00000630119.2) also transcribe from the same locus. There are two promoter-like signatures (red box) in cCREs, but one signature (dashed box) may be associated with the antisenses. PCR 0.9 kbp enhancer and Tcf21 promoter-like signiture, 1.6 kbp intragenic enhancer signatures, and the annotated TISS, then insert 0.9 kbp into Eco31I site and 1.6 kbp into BglII site of the pGL4.14. (TIF) [file pone.0309566.s001.tif]

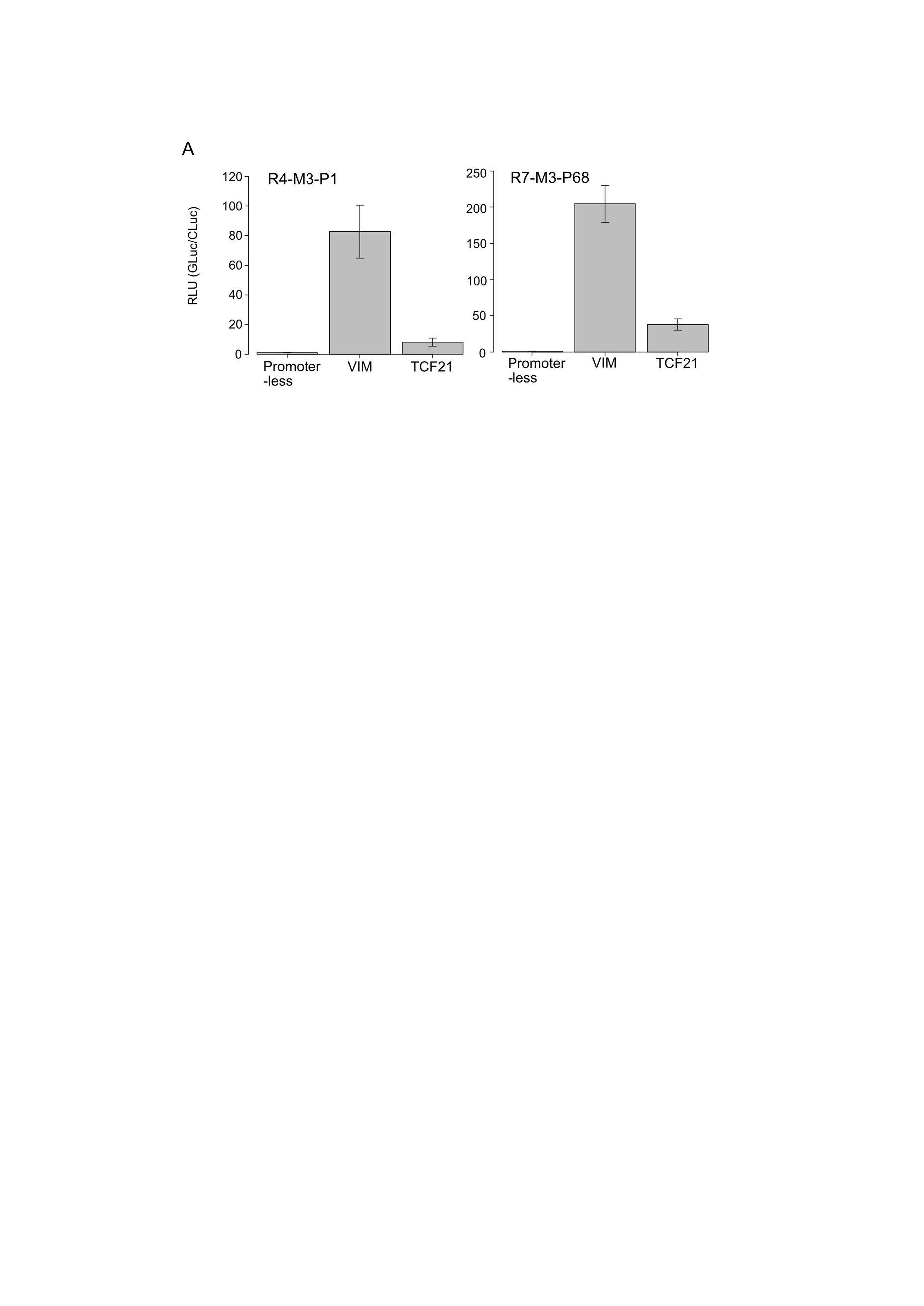

Supplement: S2 Fig — GLuc plasmid and control plasmid constitutively expressing CLuc were transduced into HCF. Luciferase activity in the medium was measured 48 hours after the transduction. Luminescence of GLuc was normalized to the luminescence of the reference CLuc (GLuc/CLuc), then the relative values with Promoter-less as 1.0 are shown. All experiments were performed in quadruplicate, bars are means, error bars are standard error of the mean. (TIF) [file pone.0309566.s002.tif]

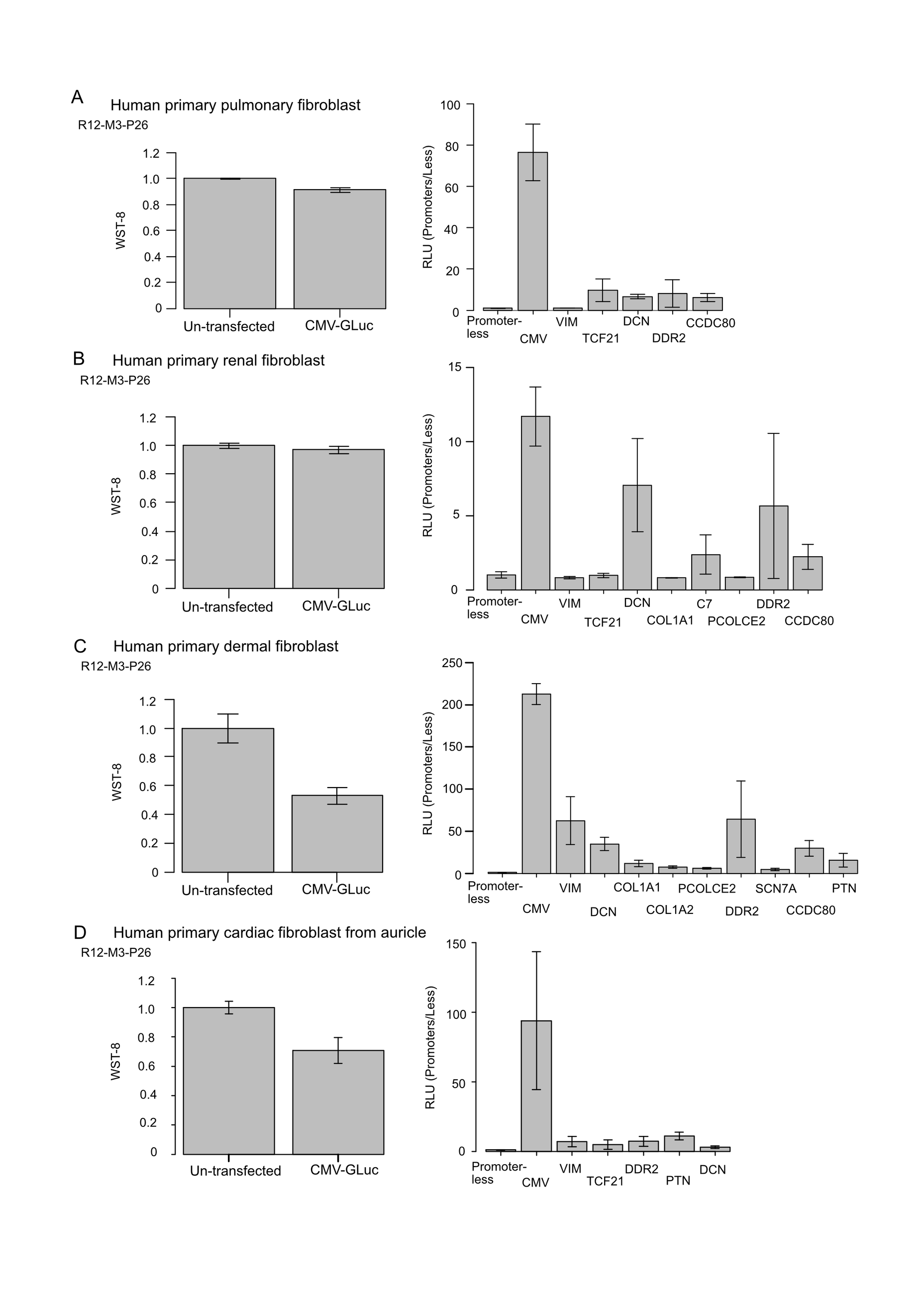

Supplement: S3 Fig — Cell viability and endogenous gene promoter/enhancer activity in primary human fibroblasts derived from lung (A), kidney (B), skin (C), and auricle of heart (D). Transfection reagent was Reagent #12, plasmids were prepared by Method #3, and the transfection protocol was Protocol #26 (R12-M3-P26; the optimal protocol in HCF). Each plasmid encodes GLuc under the control of a different enhancer/promoter sequence. Cell viability (WST-8) and luciferase activity in the medium were measured 48 hours after the transduction. Relative luminescence values are shown with promoter-less as 1.0. All experiments were performed in triplicate, bars are means, error bars are standard error of the mean. (TIF) [file pone.0309566.s003.tif]

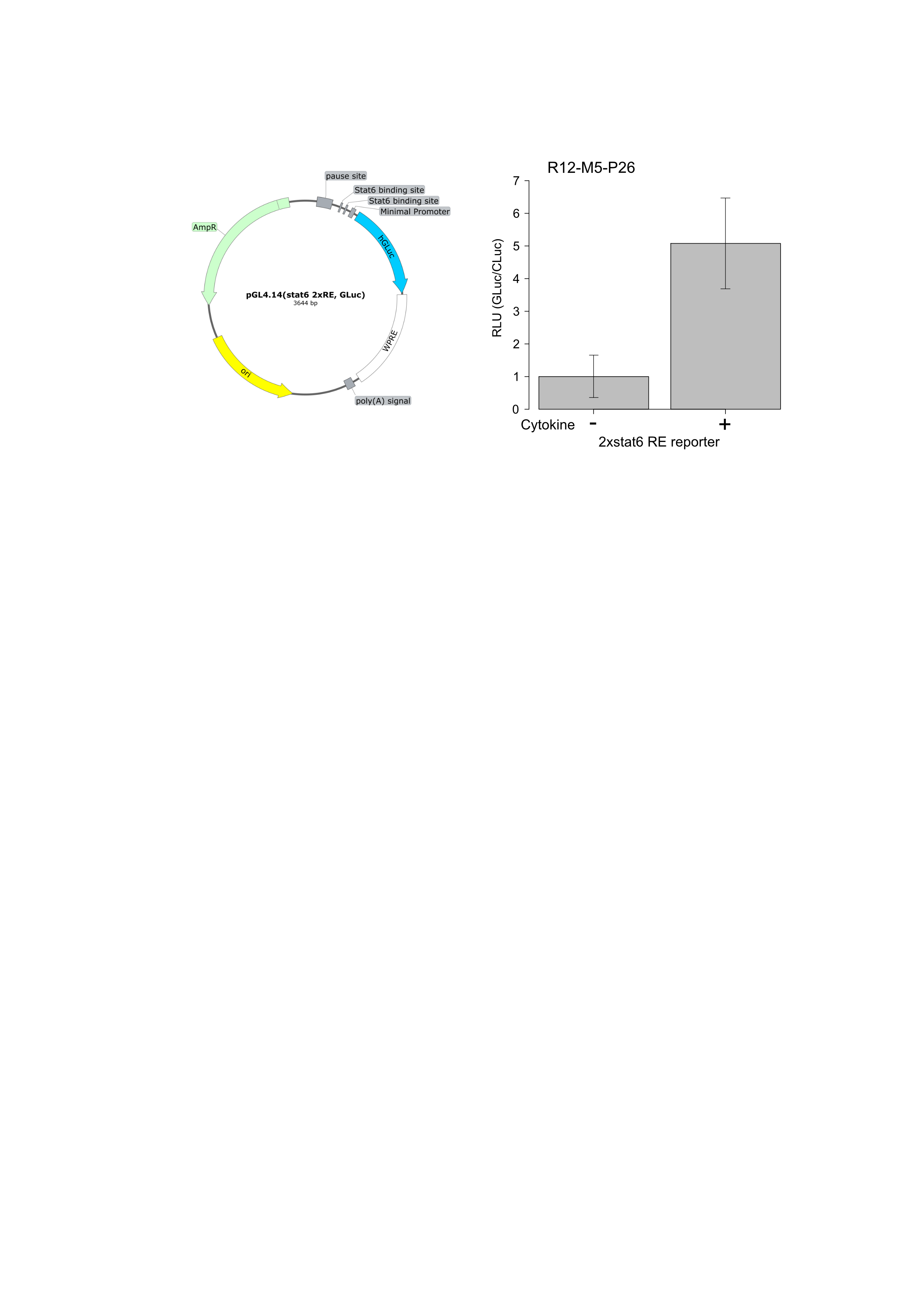

Supplement: S4 Fig — Luciferase assay measuring the activation of the JAK/STAT signal cascade in human cardiac fibroblasts (HCF) transfected with an optimized protocol using reagent 12. The plasmid contains GLuc under the control of two STAT6 binding sites upstream of a TATA box, allowing GLuc expression upon STAT6 nuclear translocation. Cytokine IL-4 was added during the initial medium change, and supernatants were collected 48 hours post-transfection for luciferase activity measurement. Relative luminescence values are shown with un-stimulated as 1.0. All experiments were performed in triplicate, bars are means, error bars are standard error of the mean. (TIFF) [file pone.0309566.s004.tiff]

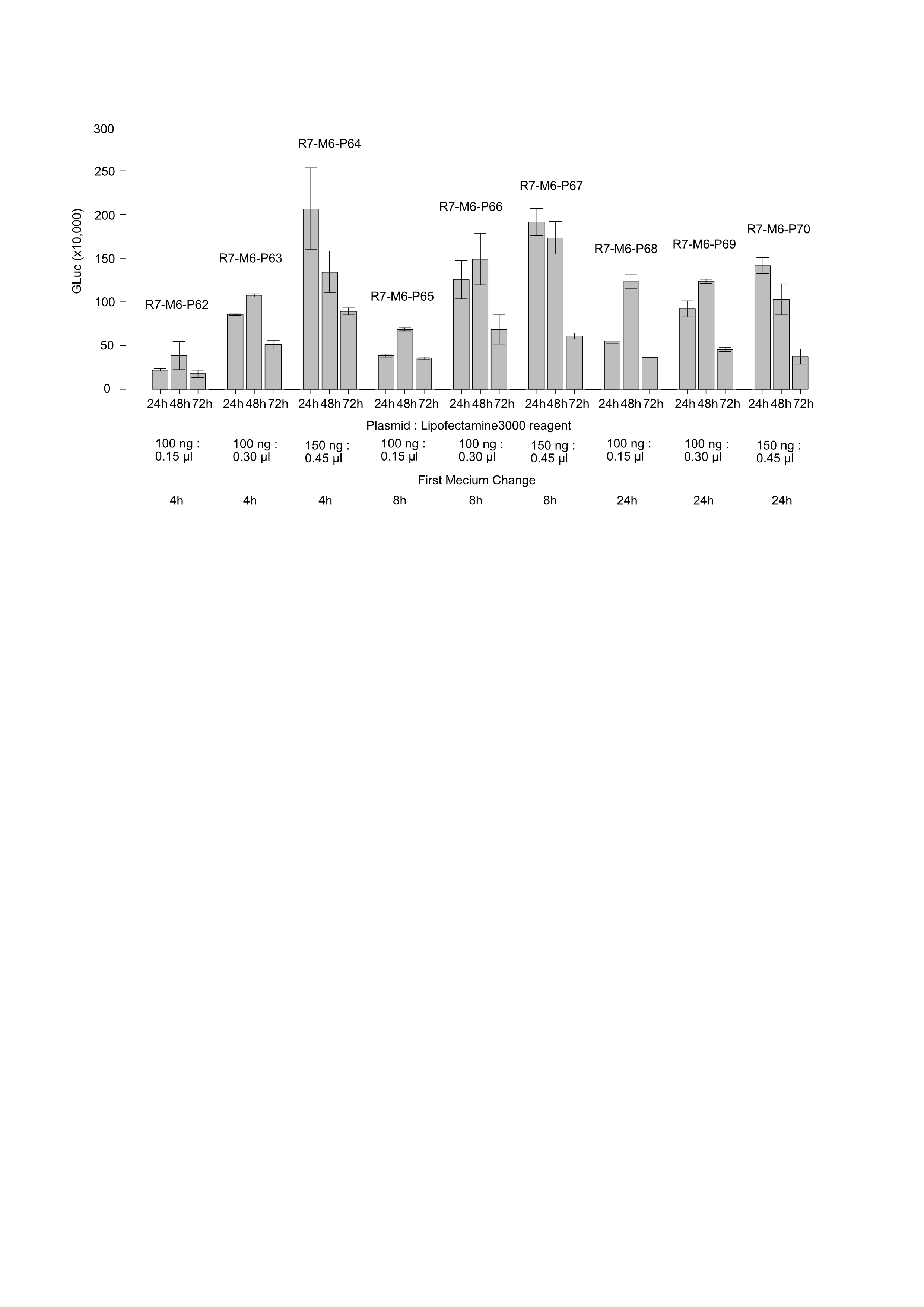

Supplement: S5 Fig — GLuc plasmid was transduced into HCF. Luciferase activity in the medium was measured 24, 48, and 72 hours after the transduction. Luminescence of GLuc was measured. All experiments were performed in duplicate, bars are means, error bars are standard error of the mean. (TIFF) [file pone.0309566.s005.tiff]

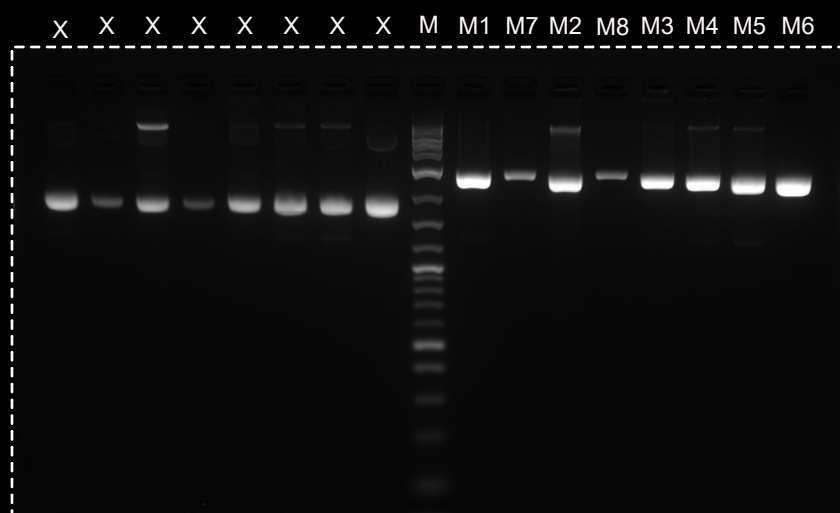

Supplement: S1 Raw images — The lanes to the left of the molecular weight marker (M) contain plasmids purified by each method, loaded at a concentration of 1000 ng/lane as measured by Nanodrop. The lanes to the right contain the plasmids after a single restriction enzyme digestion. The dotted lines indicate the outline of the agarose gel. (PDF) [file pone.0309566.s011.pdf]
