# Supplementary material for: Novel and effective plasmid transfection protocols for functional analysis of genetic elements in human cardiac fibroblasts
Source: PLoS One. 2024 Nov 26;19(11):e0309566. doi: 10.1371/journal.pone.0309566 (PMC11594401; doi:10.1371/journal.pone.0309566)
Supplement: S2 Table — All plasmids used are either transfection-grade or prepared by midiprep. (DOCX) [file pone.0309566.s007.docx]

**Supplementary Table 2. Optimal Transfection Protocols of GenomONE-GX (R4), Lipofectamine 3000 (R7), and Viafect (R12) related to Fig 5 and SFig 2.** All plasmids used are either transfection-grade or prepared by midiprep.

| GenomONE-GX (R4)'s optimal transfection into single wells in a 96-well plate | | | | | | | |
| --- | --- | --- | --- | --- | --- | --- | --- |
| Reagent 1 | Reagent 2 | Plasmid (200 ng/μl) | Reagent 5 | Reagent 4 | Incubation time | Mixture volume/well | First medium change |
|  |  |  |  |  |  |  |  |
| 0.25 μl | 1.5 μl | 1.5 μl | 1.5 μl | 1.5 μl | 5 min | 5 μl | 2 hours later |
|  |  |  |  |  |  |  |  |
| Lipofectamine3000 (R7)'s optimal transfection protocol into single well in a 96-well plate | | | | | | | |
| Lipofectamine3000/OptiMEM | | Plasmid/P3000/OptiMEM | | | Incubation time | Mixture volume/well | First medium change |
| Lipofectamine3000 | OptiMEM | Plasmid | P3000 Reagent | OptiMEM |  |  |  |
| 0.15 μl | 5 μl | 100 ng | 0.2 μl | 5 μl | 15 min | 10 μl | 24 hours later |
|  |  |  |  |  |  |  |  |
| Viafect (R12)'s optimal transfection into single well in a 96-well plate | | | | | |  |  |
| Plasmid | OptiMEM | Viafect | Incubation time | mixture volume/ well | First medium change |  |  |
| 100 ng | 10 μl | 0.6 μl | 10 min | 10 μl | 4 hours later |  |  |
|  |  |  |  |  |  |  |  |
