# Supplementary material for: Novel and effective plasmid transfection protocols for functional analysis of genetic elements in human cardiac fibroblasts
Source: PLoS One. 2024 Nov 26;19(11):e0309566. doi: 10.1371/journal.pone.0309566 (PMC11594401; doi:10.1371/journal.pone.0309566)
Supplement: Attachment — Submitted filename: Response to Reviewers_MATSUYAMA_PONE-D-24-22176.docx [file pone.0309566.s012.docx]

Response to Reviewers Manuscript ID: PONE-D-24-22176

Title: Novel and Effective Plasmid Transfection Protocols for Functional Analysis of Genetic Elements in Human Cardiac Fibroblasts

**Editor Comments:**

1. Manuscript Style Requirements

We have ensured that our manuscript now adheres to PLOS ONE's style requirements. Specifically, we revised the reference format to conform to the journal's guidelines (marked-up manuscript, page 22, line 3 to page 26, line 2).

2. Funding Statement

We amended the Funding Statement to clearly declare the commercial affiliation of the founder, Takahiro Iwamiya, and specified the role of funder in our study. The updated statement includes the following:

"The funder provided support in the form of salary for author [MM], but did not have any additional role in the study design, data collection and analysis, decision to publish, or preparation of the manuscript. The specific roles of these authors are articulated in the ‘author contributions’ section."

3. Competing Interests Statement

We have updated the Competing Interests Statement to include the commercial affiliation and affirm our adherence to PLOS ONE's policies on data and material sharing. The updated statement reads:

"I have read the journal's policy and the authors of this manuscript have the following competing interests: Takahiro Iwamiya is a cofounder and co-CEO of Metcela, Inc., and he has ownership of stocks. The corresponding author is an employee of Metcela. Takahiro Iwamiya has the authority to make payment decisions regarding employee salaries. Metcela is a company that develops VCAM1-expressing cardiac fibroblasts (VCFs) as a therapy for ischemic heart diseases. Additionally, Metcela applied for the following patents: Inventor: Takahiro Iwamiya. Assignee: Metcela Inc. Title: Composition For Injection Which Can Be Used For Treatment Of Heart Diseases And Contains Fibroblasts, And Method For Producing Fibroblast For Therapy Use. International application number: PCT/JP2018/006795. An object of the present invention is to present VCFs as a method that has not been established yet and that is useful for achieving long-term and fundamental cure of a necrotic cardiac tissue region to allow recovery of heart function.

The funder provided support in the form of salary for author [MM], but did not have any additional role in the study design, data collection and analysis, decision to publish, or preparation of the manuscript. The specific roles of these authors are articulated in the ‘author contributions’ section.

The authors declare a commercial affiliation with Metcela, Inc. This does not alter our adherence to PLOS ONE policies on sharing data and materials."

4. Data Availability

We have provided all raw data for the graphs in the Figures as Supporting Information files (S1 Table and S3 File). Therefore, the statement "All relevant data are within the manuscript and its Supporting Information files." remains accurate.

5. Data Sharing Plan

We have updated the "Additional data availability information" section, unchecking all previously indicated items. We agree to make all data freely accessible in accordance with PLOS ONE’s open data policy upon acceptance of the manuscript.

6. "Data not shown" Phrase

We have removed the phrase "data not shown" and included the relevant data as S4 Fig. The corresponding changes can be found in the marked-up manuscript (page 19, line 8) and S4 Fig.

7. Original Uncropped and Unadjusted Images

We have already provided the original gel images as S1_raw_images, complying with PLOS ONE's policy.

8. Complete and Correct Reference List

As mentioned earlier, we revised the reference format to meet PLOS ONE's style requirements, but the content of the reference list remains unchanged (marked-up manuscript, page 22, line 3 to page 26, line 2).

**Reviewer 1 Comments:**

1. Objective Clarity

We clarified the objective in the Introduction by adding a few sentences (marked-up manuscript, page 3, lines 19-29).

2. RT-qPCR Primers Table

We listed the RT-qPCR primers in a table format and clearly indicated the housekeeping genes used (marked-up manuscript, page 11, line 7 and 10).

3. Figure Legends

We followed the PLOS ONE style template, which specifies that figure captions should appear directly after the paragraph in which they are first cited.

4. Transfection Efficiency

We explicitly mentioned in the Discussion and Abstract that the efficiency of transfection using reagents R7 and R12 is less than 5% (marked-up manuscript, page 2, lines 12-15, and page 21, lines 1-3).

5. Title and Abstract Revision

We replaced "Efficient" with "Effective" in the title to better reflect our findings: "Novel and Effective Plasmid Transfection Protocols for Functional Analysis of Genetic Elements in Human Cardiac Fibroblasts." Additionally, we included the phrase "...and sufficient efficiency for functional genomic studies" at the end of the abstract to accurately convey the transfection efficiency achieved (marked-up manuscript, page 1, line 3, and page 2, lines 22-23).

**Reviewer 2 Comments:**

1. Fig 1B-D Data Presentation

We revised Fig 1C to use normalized data based on the highest group, as suggested by the reviewer. The revised figure is provided as Fig 1.

2. Missing Statement in Results Section

We deleted the incomplete statement "This suggests that..." from the results section (marked-up manuscript, page 14, line 22).

3. Statistical Comparisons in Fig 3

We included statistical comparison data in Fig 3. The revised figure is provided as Fig 3, and revised figure legend in the marked-up manuscript, page 15, lines 8-10.

4. Supplementary Table of Optimal Conditions

We added a supplementary table summarizing the optimal conditions for each reagent as S2 Table.

5. Cell Confluency Discussion

We provided a detailed discussion on cell confluency and its impact on transfection efficiency. This includes the optimal seeding density and its rationale. We also added a relevant section to the Discussion and included related data in S5 Fig (marked-up manuscript, page 19, lines 9-36, page 20, line 1, and S5 Fig).

6. HCF Source and Catalog Information

We added the catalog number and lot information for the HCF cells used in our optimization experiments (NHCF-V, LONZA, CAT #CC-2904, LOT #18TL281202) to the "Materials and Methods" section. Additionally, we performed supplementary experiments using HCFs from different donors and sources (KAC), and included these results in S3 Fig, panel D (marked-up manuscript, page 4, lines 3-15, and S3 Fig).

We hope that these revisions address all concerns and enhance the clarity and quality of our manuscript. Thank you for your consideration, and we look forward to your favorable response.

Sincerely,

MATSUYAMA Makoto, M.D., Ph.D.

Research & Development Department

Metcela Inc.

Tonomachi 3-25-16 Cybernics Medical Innovation Base, Kawasaki-ku, Kawasaki City, Kanagawa, Japan
